# Persistent At-Level Thermal Hyperalgesia and Tactile Allodynia Accompany Chronic Neuronal and Astrocyte Activation in Superficial Dorsal Horn following Mouse Cervical Contusion Spinal Cord Injury

**DOI:** 10.1371/journal.pone.0109099

**Published:** 2014-09-30

**Authors:** Jaime L. Watson, Tamara J. Hala, Rajarshi Putatunda, Daniel Sannie, Angelo C. Lepore

**Affiliations:** Department of Neuroscience, Farber Institute for Neurosciences, Sidney Kimmel Medical College at Thomas Jefferson University, Philadelphia, Pennsylvania, United States of America; Hospital Nacional de Parapléjicos, Spain

## Abstract

In humans, sensory abnormalities, including neuropathic pain, often result from traumatic spinal cord injury (SCI). SCI can induce cellular changes in the CNS, termed central sensitization, that alter excitability of spinal cord neurons, including those in the dorsal horn involved in pain transmission. Persistently elevated levels of neuronal activity, glial activation, and glutamatergic transmission are thought to contribute to the hyperexcitability of these dorsal horn neurons, which can lead to maladaptive circuitry, aberrant pain processing and, ultimately, chronic neuropathic pain. Here we present a mouse model of SCI-induced neuropathic pain that exhibits a persistent pain phenotype accompanied by chronic neuronal hyperexcitability and glial activation in the spinal cord dorsal horn. We generated a unilateral cervical contusion injury at the C5 or C6 level of the adult mouse spinal cord. Following injury, an increase in the number of neurons expressing ΔFosB (a marker of chronic neuronal activation), persistent astrocyte activation and proliferation (as measured by GFAP and Ki67 expression), and a decrease in the expression of the astrocyte glutamate transporter GLT1 are observed in the ipsilateral superficial dorsal horn of cervical spinal cord. These changes have previously been associated with neuronal hyperexcitability and may contribute to altered pain transmission and chronic neuropathic pain. In our model, they are accompanied by robust at-level hyperaglesia in the ipsilateral forepaw and allodynia in both forepaws that are evident within two weeks following injury and persist for at least six weeks. Furthermore, the pain phenotype occurs in the absence of alterations in forelimb grip strength, suggesting that it represents sensory and not motor abnormalities. Given the importance of transgenic mouse technology, this clinically-relevant model provides a resource that can be used to study the molecular mechanisms contributing to neuropathic pain following SCI and to identify potential therapeutic targets for the treatment of chronic pathological pain.

## Introduction

Spinal cord injury (SCI) is a debilitating condition with widespread symptoms that affect patient quality of life. As many as 327,000 SCI patients are currently living in the United States, and approximately 12,000 new cases are reported each year [1]. Clinical studies propose that 64–82% of SCI patients experience some form of pathological pain following injury, and this pain has been associated with mood changes as well as difficulty with work and social activities [2]. Neuropathic pain constitutes a significant percentage of pain resulting from SCI; 41% of patients encounter at-level and 34% experience below-level neuropathic pain [3] following injury. Although SCI most often involves an acute injury to the spinal cord, the resulting pathological pain is often chronic and can increase in severity over time [4]. Thus, SCI-related neuropathic pain and its treatment is an important focus of spinal cord research.

In this study, we wanted to identify the molecular changes accompanying the alterations of at-level pain sensitivity in a mouse model of cervical contusion SCI. We induced SCI in the mouse and measured changes in pain behavior, specifically thermal hyperalgesia and tactile allodynia. Additionally, we studied the expression of various proteins in the dorsal horn of the spinal cord that may contribute to neuropathic pain and the cell types expressing these proteins. Importantly, we chose to utilize a mouse model of SCI that is clinically relevant but has not previously been used to study neuropathic pain.

Currently, mouse models of SCI-induced neuropathic pain are available, but do not address cervical contusion injury. According to the National Spinal Cord Injury Statistical Center, the majority of SCI patients in the United States experience injury to the cervical regions of the spinal cord [1], but most rodent SCI models involve injury to thoracic regions. Therefore, we chose to target our SCI to the cervical region of the spinal cord.

Additionally, contusion-type injury is most common in human patients [5] and is a popular model of SCI in rodents [6]. The respiratory and other dysfunctions that commonly accompany cervical contusion injuries make this model difficult to study [7]. To date, our group [8], [9], [10] and others [11] have used models of cervical contusion SCI in the mouse and rat to study motor deficits following injury. In the study of neuropathic pain, however, contusive injury to the cervical regions of the spinal cord has not been utilized in mice. Instead, transection, compression, contusion and other procedures are used primarily at thoracic levels to induce SCI in mice for studying neuropathic pain [6]. Here, we designed a model of moderate hemicontusive injury to the cervical spinal cord in the mouse that does not develop significant respiratory or other life-threatening complications for the study of neuropathic pain.

The development of neuropathic pain following SCI involves the disruption of neurons involved in nociception in the dorsal horn of the spinal cord. Under normal circumstances, neurons in the superficial laminae of the dorsal horn are responsible for pain transmission from peripheral nociceptors to the thalamus and, ultimately, the sensory cortex. Upon injury to the spinal cord, however, local changes can occur that alter how nociceptive neurons respond to peripheral stimuli [12]. These changes, termed central sensitization, can underlie pathological pain behaviors such as allodynia and hyperalgesia [12], [13].

One mechanism involved in central sensitization is the hyperexcitability of pain transmission neurons. Neuronal hyperexcitability results in an increase in the response of neurons to electrical input or even the firing of neurons in the absence of input. Hyperexcitability is characterized by spontaneous neuronal activity, aberrant responses to subthreshold stimuli, and increased transmission of suprathreshold input [12], [13]. In SCI, pain projection neurons in the dorsal horn can become more easily excited by noxious as well as non-noxious stimuli, resulting in pathological pain transmission. Changes to the neuron itself, as well as to its extracellular environment, can alter the neuron's electrophysiological profile, increasing its resting membrane potential and/or decreasing its action potential threshold. As one example of a mechanism underlying central sensitization, increased activity of transcription factors can trigger changes in gene expression that lead to long-term alterations in neuronal function and excitability. For instance, ΔfosB, a commonly used marker of persistent neuronal activation, has been associated with plasticity of pain transmission circuits in inflammatory pain, possibly through downstream targets such as CDK5 [14], [15].

Underlying hyperexcitability and central sensitization in SCI are changes in glial activation and glutamate transporter function. Astrocytes play an essential role in the central nervous system, providing support to neurons, regulating the uptake of glutamate and other factors from the extracellular space, and modulating synapse formation and function [13], [16]. Soon after insult, astrocytes can become activated (marked by glial fibrillary acidic protein (GFAP)) and possibly even proliferative and act to minimize the effects of the injury by reestablishing the blood brain barrier, releasing antioxidants, and protecting the lesion site from detrimental molecules. Activation of astrocytes, however, can also be harmful to neighboring neurons; when persistently active, astrocytes can contribute to neuronal hyperexcitability through the release of factors such as proinflammatory molecules, nitric oxide, and ATP [13], [17], [18].

The loss of glutamate transporters by activated astrocytes and the resulting imbalance in glutamate homeostasis may also contribute to the development of post-SCI neuropathic pain. Under normal conditions, glutamate is cleared from the synapse via glutamate transporters, such as GLT1 and GLAST, located in the plasma membrane of neurons and, more often, astrocytes. However, elevated release of glutamate from presynaptic neurons and/or injured neurons and glia or from damage to the glutamate transporter system can cause an excess of glutamate to linger in synaptic and extra-synaptic locations [13]. This disruption in glutamate homeostasis leads to overactivation of glutamate receptors, which can in some instances result in increased Ca^2+^ concentrations in the postsynaptic neuron. These changes can have negative impacts on cell health and function and alter the activation state of the neuron [19], [20], [21]. In SCI, glutamate excitotoxic damage to inhibitory interneurons can also indirectly boost the excitability of nociceptive neurons in the dorsal horn [18]. In this way, the loss of GLT1 in astrocytes observed following injury by our group [22], [23], [24] and others [25], [26] may contribute to central sensitization and neuropathic pain.

Recently published data from our group showed changes in neuronal and astrocyte activation as well as GLT1 expression in the superficial dorsal horn following cervical contusion SCI-induced hyperalgesia in the rat [24]. The study characterized a moderate hemicontusion injury to cervical regions of the rat spinal cord, which resulted in ipsilateral hyperalgesic behavior. Accompanying hyperalgesia were chronic increases in ΔfosB expression in neurons, astrocyte activation and proliferation in addition to a loss of GLT1 both at the site of the injury and in intact spinal cord caudal to the contusion site [24].

In the current study we characterize increases in pain sensitivity in a mouse model of cervical contusion SCI and the accompanying molecular alterations in the dorsal horn of the spinal cord. Our model exhibits at-level thermal hyperalgesia and tactile allodynia in addition to increases in neuronal and astrocyte activation and a decrease in astrocyte glutamate transporter expression in the superficial dorsal horn. Alterations in pain behavior, as well as neuronal and glial activation and glutamate transport expression in the dorsal horn, have not previously been characterized in mice receiving cervical contusion SCI. Thus, this study provides an observation of the molecular and behavioral changes that occur following cervical SCI in the mouse that can be used to further study the mechanisms underlying neuropathic pain in SCI.

## Methods

### Animal Studies

#### Ethics Statement

All animal care and treatment were conducted in strict accordance with the *European Communities Council Directive* (2010/63/EU, 86/609/EEC and 87-848/EEC) and the *NIH Guide for the Care and Use of Laboratory Animals*. Experimental protocols were approved by the Thomas Jefferson University Institutional Animal Care and Use Committee.

#### Animals

Fifty-six male C57BL/6 mice (25–30 g; The Jackson Laboratory, USA) and 26 transgenic BAC-GLT1-eGFP reporter mice [27] were used. The BAC-GLT1-eGFP reporter mice were created by Regan et al. [27]. The transgene in these mice involves cDNA for eGFP inserted into the start codon of a mouse BAC encompassing the entire GLT-1 gene. Thus, in these mice, eGFP expression is driven by the GLT1 promoter. Mice were housed 5 per cage in a controlled light-dark environment in the Thomas Jefferson University Animal Facility and were given food and water *ad libitum*. To prevent suffering, a combination of ketamine/xylazine and isofluorane was used to anesthetize animals during surgical procedures.

#### SCI Models

We chose to utilize two models in the C57BL/6 mice in this study; one with injury at the C5 level and another with injury at the C6 level. We did this to show that contusion injury to various levels of the cervical spinal cord has the potential to produce at-level neuropathic pain. Mice were anesthetized initially with ketamine (100 mg/kg) and xylazine (5 mg/kg) injected intraperitoneally, and the anesthetic plane was maintained with 1% isofluorane for the duration of the surgery. The body was immobilized by taping the forelegs to a fixation plate, and the head was immobilized by the isofluorane nose cone. The skin and muscle overlying the spinal column were retracted from levels C3 to T1, and spinal cord was exposed by unilateral laminectomy from the midline blood vessel to the lateral edge of the vertebral lamina at the level of C5 or C6. The spinal column was stabilized at the C3 and T1 spinous processes by microforceps attached to the fixation plate and the far right and left corners. C57BL/6 mice received unilateral contusion injuries at the exposed portion of the spinal cord using the Infinite Horizon Impactor (Precision Systems and Instrumentation; Lexington, KY) with an impactor tip of 0.7 mm in diameter, a force of 40 kilodynes, two seconds of dwell time, and an approximately perpendicular impact angle, parameters similar to our previously published cervical contusion SCI paradigms [8], [9], [10]. Injury parameters for BAC-GLT1-eGFP mice included a 1.0 mm diameter tip, 50 kilodyne force, and no dwell time at the C5 level. Control animals received unilateral laminectomy but not contusion injury to the spinal cord. Following injury, the forceps were removed and the muscle layers were secured with a sterile 4-0 silk suture. Sterile wound clips were used to close the skin, and 1 ml of lactated Ringers solution and 0.1 mg/kg of buprenorphine-HCl were administered subcutaneously.

#### Unilateral Hargreaves Thermal Test

A modified version of the Hargreaves test for thermal hyperaglesia, based on previously established methods [28], was conducted for the forepaws of each animal. This test detects sensitivity to thermal nociceptive stimuli by determining the latency to withdrawal of the paw from an infrared stimulus of a particular intensity. Prior to surgery, baseline measures were collected once weekly for two weeks. Following contusion SCI or laminectomy, each animal was tested weekly for six weeks. Before the first baseline test, the animals were acclimated to the testing room for an hour each day for five days. Prior to each session, mice were also acclimated to the testing room for an hour. Individually, the mice were restrained manually by the scruff only and placed on a thin glass pane, with one of the forepaws directly above the source of the infrared stimulus (UgoBasile; Comerio, VA). The animals continued to be restrained, but movement of the forepaw was unimpeded. The stimulus was initiated, and a fiber optic sensor on the movable infrared heat source measured the time to forepaw withdrawal. Forepaw withdrawal was defined as a quick movement of the paw away from the infrared stimulus often accompanied by licking of the forepaw. Spontaneous movements of the forepaw were not considered forepaw withdrawal and resulted in discarding the data and repeating the trial. Three trials were conducted for each forepaw of each animal, alternating the left and right forepaws, with an inter-trial interval of 120 seconds.

#### von Frey Filament Test

Semmes-Weinstein monofilaments (Stoetling Company; Dale, IL) ranging from 1.65 grams to 4.56 grams of force were utilized to measure tactile allodynia using the up-down method. Testing occurred twice prior to surgery to obtain baseline data and once weekly for six weeks after injury. Acclimation procedures were the same as those used for the Hargreaves test. Each mouse experienced ten trials per testing day with an inter-trial interval of 120 seconds. The first trial of each testing day utilized a filament of 3.84 grams. A single trial involved directing a monofilament at the center of the plantar surface of the forepaw of interest and application until the filament buckled. Mice were not restrained for this testing. The response of the mouse was then recorded as a positive or negative withdrawal response. The filament application was considered to produce a positive response if the mouse rapidly withdrew its forepaw, which was mostly accompanied by vocalization and/or licking of the forepaw. If the response was positive, the filament used in the next trial would be the next smaller filament. If instead the response was negative, the next larger filament would be used in the next trial. Withdraw threshold was determined as the lowest filament/force that evoked a positive withdrawal response in greater than 50% of the trials with that particular filament.

#### Grip Strength Testing

Grip strength was measured by the DFIS-2 Series Digital Force Gauge (Columbus Instruments, OH) [26], used previously by our group [8]. Attached to the force gauge is a triangular metal pull bar that allows for the transduction of force from the mouse to the gauge. The Digital Force Gauge measures the strength with which the mouse is able to grasp and hold onto a thin metal bar. Three baseline measures spread across two weeks were obtained prior to surgery, and mice were tested once weekly for six weeks following injury. In each trial, the mouse was allowed to grab the bar with one forepaw and was then quickly pulled away from the gauge so its grip was released, providing a measurement of the force with which the mouse gripped the bar. During each testing session, three trials for each forepaw were performed with an inter-trial interval of at least 60 seconds.

### Histological Analyses

#### Tissue Processing

Mice were sacrificed at two days or two or six weeks following injury or laminectomy by anesthetic overdose and transcardial perfusion with 0.9% saline followed by 4% paraformaldehyde. The spinal cord was harvested following perfusion, fixed in 4% paraformaldehyde at room temperature for 24 hours, washed in 0.1M phosphate buffer at 4°C for 24 hours and then cryoprotected in 30% sucrose in 0.1M phosphate buffer at 4°C for three days. The cervical/rostral thoracic spinal cord was dissected from the rest of the cord, embedded in freezing medium, and flash-frozen with dry ice. Embedded tissue was cut transversely by cryostat at a thickness of 30 µm and mounted directly onto slides. Slides were stored at −20°C.

#### Motor Neuron Counts

A representative sample (i.e. every fifth section) of spinal cord tissue was used to locate the injury epicenter. Slides were thawed at room temperature for an hour and stained with 0.5% Cresyl violet acetate/Eriochrome cyanine. For each section, large motor neurons ventral to the central canal in the grey matter were counted for the ipsilateral and contralateral sides [9]. The motor neuron cell bodies were identified by their size and characteristic morphology. The injury epicenter was defined as the section with the fewest large motor neurons in the ipsilateral ventral grey matter.

#### Immunohistochemistry

**Figure 1:**
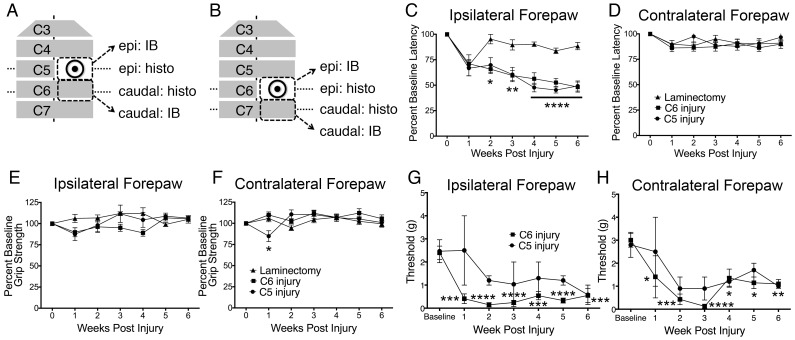
Animals receiving unilateral cervical contusion SCI exhibited persistent thermal hyperalgesia and tactile allodynia in the forepaw. Cervical contusion SCI was administered at the C5 or C6 level of the spinal cord while uninjured control animals received only laminectomy at the C6 level. Tissue from the level of laminectomy or epicenter of the injury and from the region immediately caudal was harvested for either immunoblotting or histology (A–B). Thermal hyperalgesia was measured using a modified version of the Hargreaves test. Animals receiving contusion injury showed a decrease in latency to withdrawal in the ipsilateral forepaw, measured as a percentage of baseline latency, compared to control animals (C). This difference was first observed at two weeks after injury and persisted until the animals were sacrificed at six weeks after injury. No change in withdrawal latency was seen in injured vs. uninjured animals in the contralateral forepaw (D). Animals receiving SCI were also tested for tactile allodynia using von Frey filament testing. In animals receiving C6 injury, but not C5 injury, a significant decrease compared to pre-injury baseline in the force threshold required to elicit a withdrawal response was evident both ipsilaterally (E) and contralaterally (F) for each of the six weeks following injury. The decrease in ipsilateral forepaw withdrawal latency and bilateral force threshold in injured animals occurred in the absence of changes in grip strength in either forepaw (G–H). Epi = epicenter; IB = immunoblotting; histo = histology; * = p<0.05; ** = p<0.01; **** = p<0.0001.

Immunohistochemical analysis was performed both ipsilaterally and contralaterally to the unilateral injury at the injury epicenter as well as 1.05 mm caudal to the epicenter (Figure 1A–B). Sectioned tissue was thawed and dried at room temperature for an hour and then washed in TBS. Sections were blocked in 10% normal goat serum/0.2% Triton/TBS at room temperature for one hour followed by primary antibody incubation in 2% goat serum/0.5% Triton/TBS at 4°C overnight. Secondary antibody was incubated in 2% goat serum/0.5% Triton/TBS at room temperature for two hours. Primary and secondary antibodies used were rabbit polyclonal ΔfosB (IHC 1∶100; Santa Cruz, USA) [30], rabbit polyclonal Ki67 (IHC 1∶200; Abcam, USA) [31], mouse monoclonal GFAP (IHC 1∶400, Sigma Aldrich, USA) [32], rabbit polyclonal GLT1 (IHC 1∶800, kindly provided by Jeffrey Rothstein's lab at Johns Hopkins University) [29],, mouse monoclonal CD11b (IHC 1∶4,000, AbD Serotec, USA), rhodamine-conjugated goat-anti-rabbit IgG (1∶100; Jackson Immuno, USA) and FITC-conjugated goat-anti-mouse IgG (1∶100; Jackson Immuno, USA).

#### Fluorescence Imaging and Quantification

**Figure 2:**
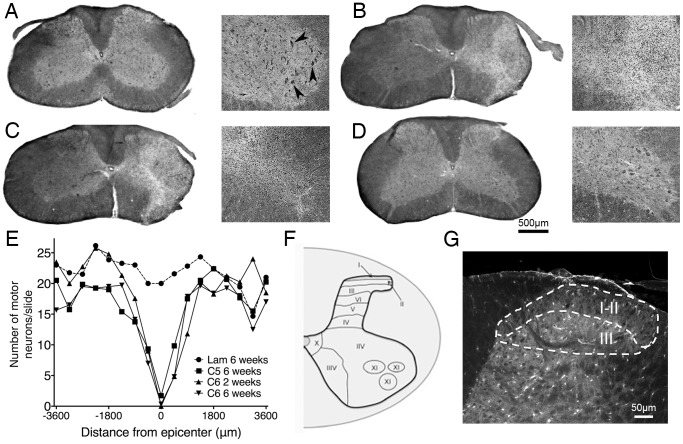
Unilateral contusion SCI induced a loss of ventral horn motor neurons at the injury epicenter. Harvested tissue was stained with Cresyl violet and Eriochrome cyanine. At the level of the laminectomy, uninjured control animals (A) exhibited large motor neurons in the ventral horn (arrowheads). At six weeks post-injury, animals receiving unilateral C5 (B) or C6 (C) contusion SCI showed a loss of these motor neurons at the injury epicenter but not 1.0 mm caudal to the injury (D). The spread of ventral horn motor neuron loss was approximately 1.0 mm rostrally and 1.0 mm caudally (2.0 mm total) from the epicenter (E). Immunohistochemical analyses of the injury models were performed in laminae I–II and lamina III of the cervical spinal cord dorsal horn (F–G). Lam = laminectomy.

Imaging and quantification of fluorescence immunostaining and BAC-GLT1-eGFP tissue were performed using a Zeiss Imager M2 upright fluorescence microscope [23]. Images were taken and analyses performed on saved PNG images for analyses. All immunostaining was quantified in the most superficial laminae (I–II) or lamina III of the spinal cord dorsal horn (Figure 2F–G). Laminae I–III were delineated by beginning at the most lateral portion of the dorsal horn, drawing a horizontal line across the gray matter, and following the outline of the gray matter back to the lateral extension of the dorsal horn. The border between laminae I–II and lamina III was identified by the change in tissue morphology represented by darker tissue in laminae I–II and lighter tissue in lamina III. Quantification analyses were performed using Metamorph software (Molecule Devices; Sunnyvale, CA). ΔfosB- and Ki67-positive cells were quantified by counting the stained nuclei in the superficial laminae. GFAP and GLT1 staining was more diffusely distributed and was therefore quantified in laminae I–II as an integrated intensity, or the sum of the intensity of pixels over a region, at constant exposure, brightness, and contrast. In BAC-GLT1-eGFP tissue, quantification was performed for laminae I–II and lamina III. eGFP-positive cells were counted separately for these regions [23].

### Biochemical Analyses

#### Tissue Harvesting

Sacrifice occurred at two or six weeks after injury or laminectomy. Following anesthetic overdose, animals were perfused with 0.9% saline and the cervical spinal cord removed, dissected, and flash-frozen in dimethylbutane. The spinal cord was sub-dissected to collect the ipsilateral cord at the level of the injury and the segment directly caudal to the injury (Fig. 1A–B). To identify the regions of interest, the muscles were first removed to expose the spinal column. The laminectomy area was located, and the contralateral vertebral lamina was removed. Microdissection scissors were used to cut the spinal cord along the midline, and a 1.0 mm piece of tissue between the remaining laminae was collected. Flash-frozen tissue was stored at −80°C.

#### Western Blotting

Tissue samples were homogenized on ice in 50 µl of RIPA buffer containing 50 mM TRIS-HCl pH 7.6, 150 mM NaCl, 2 mM EDTA, 0.1% SDS, 0.01% NP-40, and Protease Inhibitor Cocktail (Roche Diagnostics, Indianapolis, IN). Protein concentration was determined by the Bradford assay, and equal amounts of protein were run on 4–12% Bis-Tris gels and transferred to nitrocellulose membranes. Odyssey blocking buffer (Li-Cor; Lincoln, NE) was used to block the membranes at room temperature for one hour. Primary antibodies for GLT1 (1∶2,000) and actin (1∶2,000; Abcam) were diluted in Odyssey blocking buffer and membranes were incubated at 4°C overnight. The membranes were then probed with IRDye-conjugated goat anti-rabbit or goat anti-mouse IgG (1∶20,000; Li-Cor) at room temperature for one hour. Imaging was performed by the Li-Cor Odyssey infrared imaging system, and GLT1 band intensity was measured and normalized to actin band intensity using ImageJ software [22].

### Statistical Analyses

All data are presented as mean ± SEM. Statistical analyses were performed using GraphPad Prism (GraphPad Software, Inc.; La Jolla, CA). Bar graphs are presented as averages with error bars representing standard error. The Hargreaves test and von Frey threshold data were analyzed using a two-way ANOVA with repeated measured to compare the means within each group (laminectomy, C5 injury, or C6 injury) at each time point. The immunohistochemical and biochemical data were assessed with one-way ANOVA, comparing each group (laminectomy, C6 2 weeks, or C6 6 weeks) to one another. Statistical significance is defined as p<0.05 for all analyses.

## Results

### Robust and persistent forepaw thermal hyperalgesia and tactile allodynia were observed following unilateral cervical contusion SCI

Here we present a model of unilateral cervical contusion SCI in the mouse that exhibits persistent changes in pain behavior in the absence of motor deficits. Following baseline behavior testing, animals received unilateral laminectomy and contusion injury of 40 kilodyne of force with two seconds of dwell time at either the C5 (Fig. 1A) or C6 (Fig. 1B) level of the spinal cord. Control animals underwent laminectomy without injury. In both injury models, no grossly observable deficits in respiratory or other vital functions were present.

Beginning one week after injury, thermal hyperalgesia, tactile allodynia and grip strength were measured in laminectomy (n = 8) and injured animals (C5: n = 8; C6: n = 10) for six weeks. A modified version of the Hargreaves test was utilized to measure sensitivity to noxious thermal stimuli in the ipsilateral and contralateral forepaws. In both injured and uninjured animals, forepaw withdrawal upon noxious thermal stimulation was deliberate and was often accompanied by licking or scratching of the paw. Two-way ANOVA was used to compare withdrawal latency between animals with C5 injury, C6 injury, and laminectomy only at each time point. Post-injury measurements of latency to forepaw withdrawal are reported as a percentage of the baseline average for each animal. In laminectomy-only animals, withdrawal latency non-significantly decreased in the ipsilateral forepaw at one week post-injury but returned to and persisted at baseline levels until six weeks after injury (Fig. 1C). Beginning two weeks after injury and persisting for the length of testing, ipsilateral forepaw withdrawal latencies for animals receiving unilateral contusion SCI at the C5 or C6 level were significantly reduced compared to uninjured laminectomy animals (Fig. 1C). In the contralateral forepaw, no significant changes amongst the three experimental groups were seen (Fig. 1D).

In addition, tactile allodynia was assessed in C5 injured and C6 injured animals. The threshold force required to elicit a withdrawal response in each of the forepaws was measured by the von Frey test. In the C6 injury group, but not as robustly in the C5 injury group, tactile allodynia was evident (Fig. 1E–F). Animals were tested prior to injury to obtain baseline data and were then assessed for six weeks following injury. When values for each testing week were compared to pre-injury baseline data, animals receiving C6 injury exhibited a significant decrease in threshold force both ipsilaterally (Fig. 1E) and contralaterally (Fig. 1F). Although the data were not significant for animals receiving C5 injury, there was a similar trend in decreased response threshold.

While thermal and mechanical sensitivity increased in injured animals, this was not associated with changes in grip strength. Grip strength results were analyzed by two-way ANOVA to identify significance between each group at each time point. For the ipsilateral forepaw, no changes were observed between injured and uninjured animals for the six weeks following surgery (Fig. 1G). A significant decrease in contralateral grip strength for animals receiving injury at the C5 level was seen one week after injury compared to laminectomy or C6 injury (Fig. 1H). However, because the change occurred so soon after surgery and grip strength recovered to baseline levels by the next testing session, a spinal shock mechanism may be responsible for this unexpected change.

Together, the Hargreaves, von Frey, and grip strength data suggest hypersensitivity to noxious thermal and tactile stimuli in the absence of altered grip strength, indicating that the observed changes in forepaw withdrawal resulted from aberrant pain processing rather than motor deficits.

### Unilateral cervical contusion SCI produced an injury characterized by a focal loss of ventral horn motor neurons

The animals that received unilateral contusion SCI exhibited loss of large motor neurons in the ventral grey matter as well as a disruption of lateral grey matter, dorsolateral funiculus, and ventrolateral funiculus of the ipsilateral hemicord (Fig. 2B–C). In laminectomy-only animals (n = 8), large motor neurons of distinct morphology were present throughout the ipsilateral ventral horn (Fig. 2A). These motor neurons were absent or their numbers were greatly reduced, however, at the injury site in animals receiving C5 (Fig. 2B; n = 8) or C6 (Fig. 2C; 2 weeks: n = 8; 6 weeks: n = 10) injury. This effect was limited to the injury site, as motor neuron populations and white matter were intact in regions rostral and caudal to the injury (Fig. 2D). Additionally, no loss of motor neurons was observed on the contralateral side of the spinal cord at any point along the rostral-caudal axis (data not shown). Thus, the anatomical disruption sustained by injured animals was restricted to the injury site ipsilaterally.

In order to quantify the contusion injury sustained by the animals and identify the injury epicenter along the rostral-caudal axis, sections were stained with Cresyl violet acetate/Eriochrome cyanine and motor neurons in the ventral horn were counted. The epicenter of the injury was determined by the extent of motor neuron loss, and the motor neuron counts were plotted by distance from this site (Fig. 2E). Significance was determined using one-way ANOVA to compare motor neuron counts between each group. Compared to laminectomy animals, a significant decrease in ventral horn motor neurons was observed for animals receiving injury at either cervical level and at both time points. The spread of both C5 and C6 injuries was approximately 2.0 mm (1.0 mm rostrally and 1.0 mm caudally from the epicenter) with a gradual decrease in motor neurons immediately rostral and caudal to the epicenter. The epicenter identified by this method was used for further immunohistochemical analyses of the neuronal and glial populations of the spinal cord dorsal horn, which were performed in laminae I–II and laminae III (Fig. 2F–G).

### Chronic neuronal activation in the superficial laminae of the dorsal horn resulted from contusion SCI

Because injury at either the C5 or C6 level produced thermal hyperalgesia and tissue damage to a similar extent, we chose to move forward with a single model, the C6 injury, for further analyses. ΔfosB, a truncated splice version of the immediate early gene c-fos, is a transcription factor and marker of persistent neuronal activation. Chronic upregulation of this gene has been implicated in plasticity and inflammatory pain [14], [15] and has the potential to contribute to neuropathic pain. To study the extent of ΔfosB expression in our SCI model, we sacrificed animals at two weeks and six weeks after injury (n = 7–10) or six weeks after laminectomy (n = 8). Immunohistochemistry for ΔfosB was performed at the injury epicenter and 1.0 mm caudal to the injury in laminae I–II.

**Figure 3:**
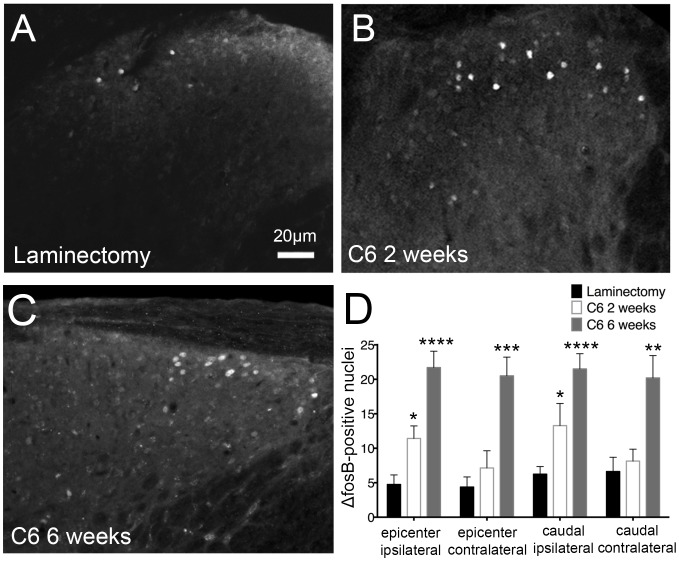
Chronic neuronal activation in the dorsal horn resulted from cervical contusion SCI. ΔfosB staining was used to measure the extent of persistent neuronal activation in the spinal cord. Following injury, ΔfosB-positive nuclei (arrowheads) were evident in laminae I–II (A–C). At all regions, laminectomy control animals (A) showed little-to-no ΔfosB expression (D). Two weeks after C6 injury (B), a significant increase in numbers of ΔfosB expressing cells in the superficial laminae of the ipsilateral dorsal horn was observed at the injury epicenter and caudal to the injury (D). ΔfosB levels were further increased at all regions in animals sacrificed six weeks post-injury (C, D). * = p<0.05; ** = p<0.01; *** = p 0.001; **** = p<0.0001.

We found that ΔfosB-positive cells exhibited a defined pattern of nuclear staining in the superficial laminae. In our SCI model, we observed a persistent increase in the number of Δfos-expressing cells, suggesting chronic neuronal activation following cervical contusion injury. One-way ANOVA was used to compare the number of ΔfosB-positive cells between each group. Following laminectomy, limited-to-no ΔfosB expression was seen in the dorsal horn (Fig. 3A). Two weeks after injury (Fig. 3B), there was an increase in ΔfosB-positive cells on the ipsilateral side at and caudal to the injury epicenter. At six weeks (Fig. 3C), ΔfosB was upregulated bilaterally in the superficial laminae at the injury epicenter and 1.0 mm caudal. These changes are quantified in Figure 3D. The greatest increase in ΔfosB expression was observed at six weeks, suggesting that its expression was persistent and cumulative over time. Additionally, the expression of ΔfosB in the injured animals was specific to the dorsal horn of the spinal cord. There were few to no ΔfosB-positive cells in other regions of the spinal cord, including the ventral horn. This suggests that the effects of our injury paradigm on chronic neuronal activation were focal to the dorsal horn, an important region of the spinal cord for pain transmission and modulation.

### Astrocytes were activated and proliferated in the dorsal horn of the spinal cord following cervical contusion SCI

Astrocytes play important protective and homeostatic roles in both the immediate and delayed response to CNS injury. Chronic activation in the spinal cord, however, can contribute to neuronal hyperexcitability of pain transmission neurons that underlies neuropathic pain [12], [13]. When astrocytes become activated, they express distinct proteins and in some cases proliferate [21]. In order to measure the spatial and temporal change in astrocyte activation in our model of SCI, we used immunohistochemistry to stain for GFAP, expressed by activated astrocytes, and Ki67, a marker of cellular proliferation. As in the previous immunohistochemical analyses, animals were studied two or six weeks after injury (n = 7–10) or six weeks after laminectomy (n = 8). For each analysis, one-way ANOVA was utilized to identify significance between the three groups.

**Figure 4:**
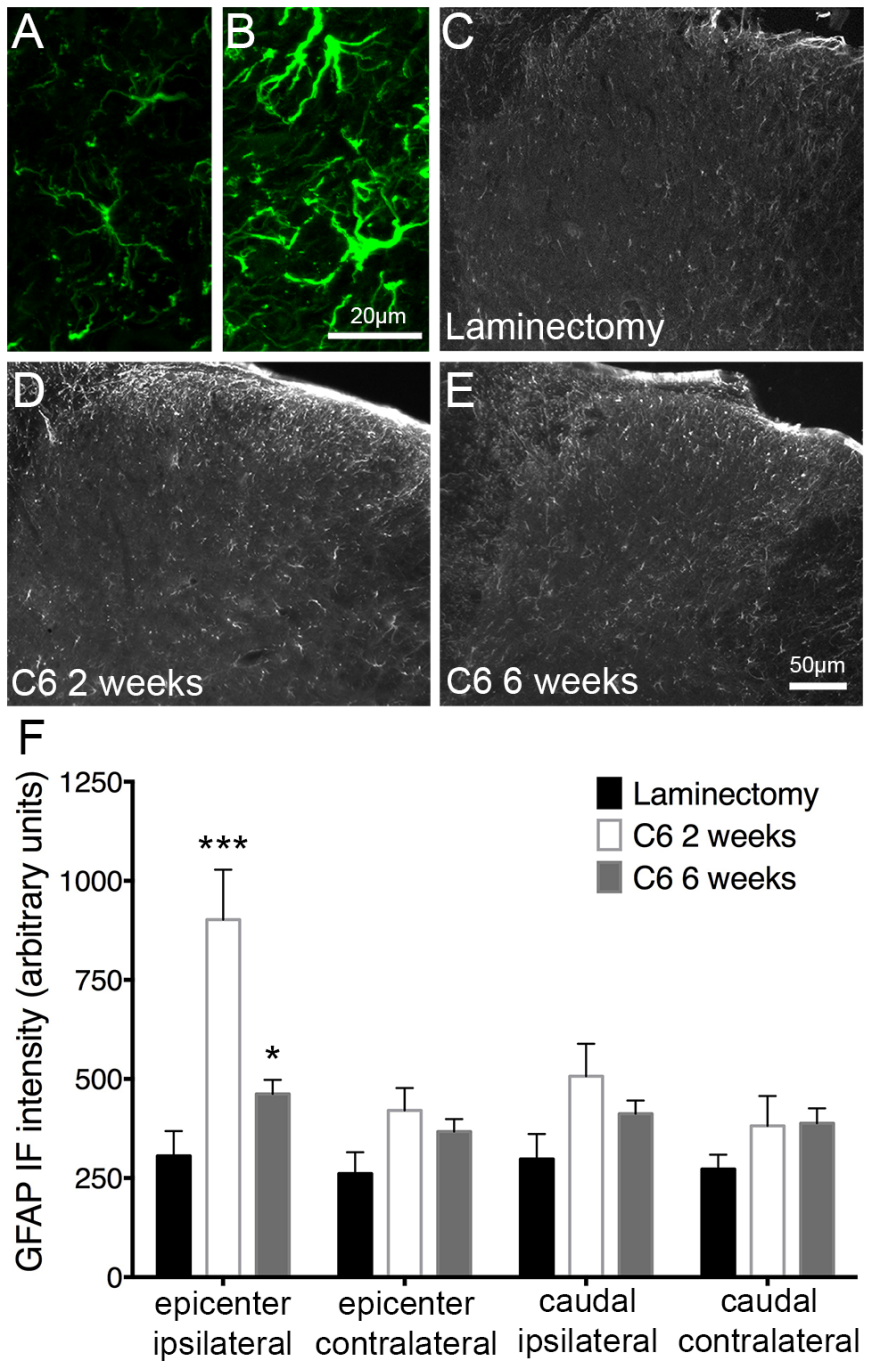
Astrocytes were activated and proliferated in the dorsal horn following cervical contusion SCI. Astrocyte activation in the superficial dorsal horn was characterized by quantification of GFAP expression in laminae I–II. (A) and (B) show representative images of dorsal horn GFAP expression at high magnification in laminectomy and injured animals, respectively. At the injury epicenter, compared to uninjured control animals (C), an increase in GFAP expression in the ipsilateral dorsal horn was evident at two (D) and six weeks (E) following injury. No significant changes in GFAP expression were observed at the other regions studied (F). IF = immunofluorescence; * = p<0.05; *** = p<0.001.

Figure 4B shows the distinct morphology of activated astrocytes observed following injury compared to uninjured control (Fig. 4A). We found that injury induced diffuse GFAP expression throughout the gray matter of the spinal cord; therefore, we quantified astrocyte expression in laminae I–II by measuring GFAP intensity. Compared to laminectomy (Fig. 4C), GFAP intensity was significantly greater at both time points in animals receiving contusion SCI (Fig. 4D–E). These changes were observed ipsilaterally at the injury epicenter, with no alterations in GFAP expression at the other regions analyzed (Fig. 4F). GFAP expression was highest two weeks after injury, which suggests that, while post-injury astrocyte activation is chronic, it may lessen over time.

**Figure 5:**
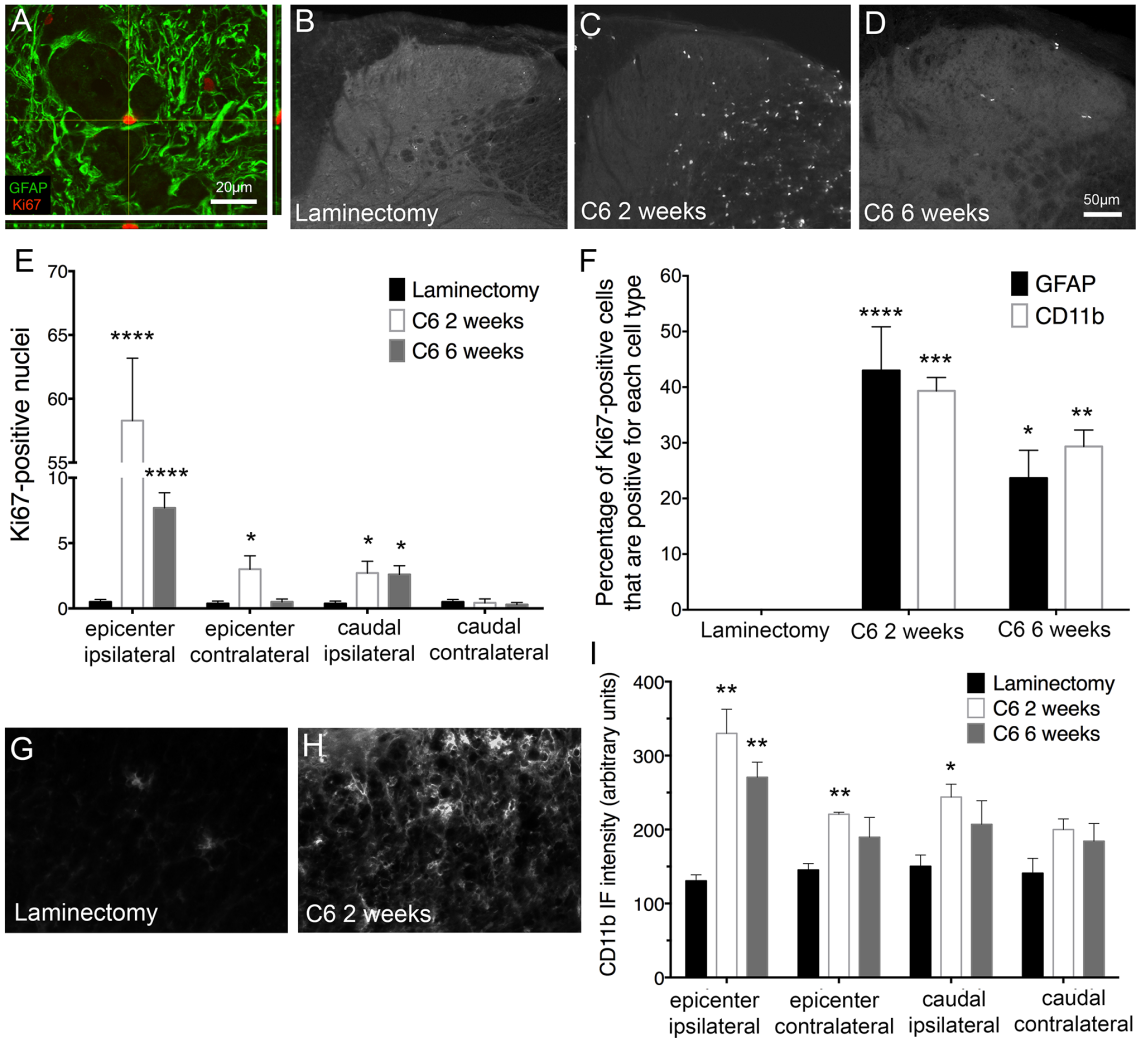
Enhanced cell proliferation, including proliferation of astrocytes, was evident after cervical contusion SCI. In the superficial laminae of the injured ipsilateral dorsal horn, we observed cells co-expressing GFAP and Ki67 (A), representing activated and proliferating astrocytes. In laminectomy control animals, little to no cell proliferation was evident (B). However, the number of Ki67-positive cells was significantly increased at two weeks (C) and six weeks (D) after injury on the ipsilateral side both at the level of and caudal to the injury (E). Additionally, at two weeks, there was a significant increase in proliferating cells contralaterally at the injury site (E). At both two and six weeks, a significant percentage of Ki67-positive cells were also either GFAP-positive or CD11b-positive (F). Compared to laminectomy (G), the intensity of CD11b expression in the superficial dorsal horn was greater in animals with C6 SCI at both two (H–I) and six (I) weeks after injury. This increase was significant at the injury site both ipsilaterally and contralaterally, as well as caudal to the injury on the contralateral side (I). * = p<0.05; ** = p<0.01; **** = p<0.0001.

We next sought to study the extent of astrocyte proliferation in the dorsal horn to further support our assertion that astrocytes respond to the spinal cord insult we induced. We confirmed using confocal microscopy that, after injury, the majority of Ki67-expresing cells in the superficial were GFAP-positive astrocytes (Fig. 5A). Basally, proliferating cell numbers, and thus Ki67 expression, in the spinal cord were low (Fig. 5B). Two weeks following injury (Fig. 5C), the superficial laminae expressed a robust increase in bilateral Ki67 that was also seen in the ipsilateral dorsal horn caudal to the injury (Fig. 5E). At six weeks (Fig. 5D), an increase in Ki67 was also observed but did not reach the levels seen at two weeks.

We identified the phenotypes of these Ki67-positive proliferative cells. First, we quantified microglial activation in the superficial dorsal horn by immunostaining for CD11b (Fig. 5G–I). Like astrocytes, microglia play a role in both immediate and delayed responses to injury to the spinal cord and may contribute to the development of pathological pain (13). Compared to laminectomy (Fig. 5G), the intensity of CD11b expression was greater in animals with C6 SCI at both two (Fig. 5H–I) and six (Fig. 5I) weeks after injury. This increase was significant at the injury site both ipsilaterally and contralaterally, as well as caudal to the injury on the ipsilateral side (Fig. 5I).

We also quantified the percentage of Ki67-positive cells that were either GFAP-positive or CD11b-positive. This allowed us to determine if the proliferative cells are, in fact, astrocytes (GFAP-positive) or microglia (CD11b-positive). One-way ANOVA was used to identify significance in Ki67 and GFAP or CD11b colocalization. In laminectomy animals, the percentage of cells that are positive for both Ki67 and GFAP or CD11b is zero because there are no Ki67-positive cells present (Fig. 5F). At both two weeks and six weeks after C6 injury, there is a significant increase in the percentage of Ki67-positive cells that are also GFAP-positive or CD11b-positive (Fig. 5F). No significant difference exists between the percentage of Ki67-positive cells that are GFAP-positive or those that are CD11b-positive (Fig. 5F). Thus, both astrocytes and microglia are proliferative following cervical contusion SCI. These data further support the notion that activated astrocytes are present in the spinal cord following injury but retreat over time and/or that proliferation is mostly an early process of astrocyte activation post-SCI. The effects of astrocyte activation can be lasting, however, through the release of factors, for example, that can contribute to hyperexcitability.

### Cervical contusion SCI led to reduced expression of GLT1 in the superficial dorsal horn of the spinal cord

The most abundant glutamate transporter in the CNS is GLT1, and its greatest expression is in astrocytes [33]. Through GLT1 and other glutamate transporters, astrocytes clear excitatory amino acids from the synapse after they are released, regulating glutamate's duration of action, controlling normal synaptic communication, and protecting cells from excitotoxic effects [33]. After SCI, we and others [23], [24], [25], [26] have reported a loss in astrocyte GLT1 expression. In this study, we utilized two animal models to study localized decreases in GLT1 in the context of neuropathic pain in the superficial dorsal horn.

**Figure 6:**
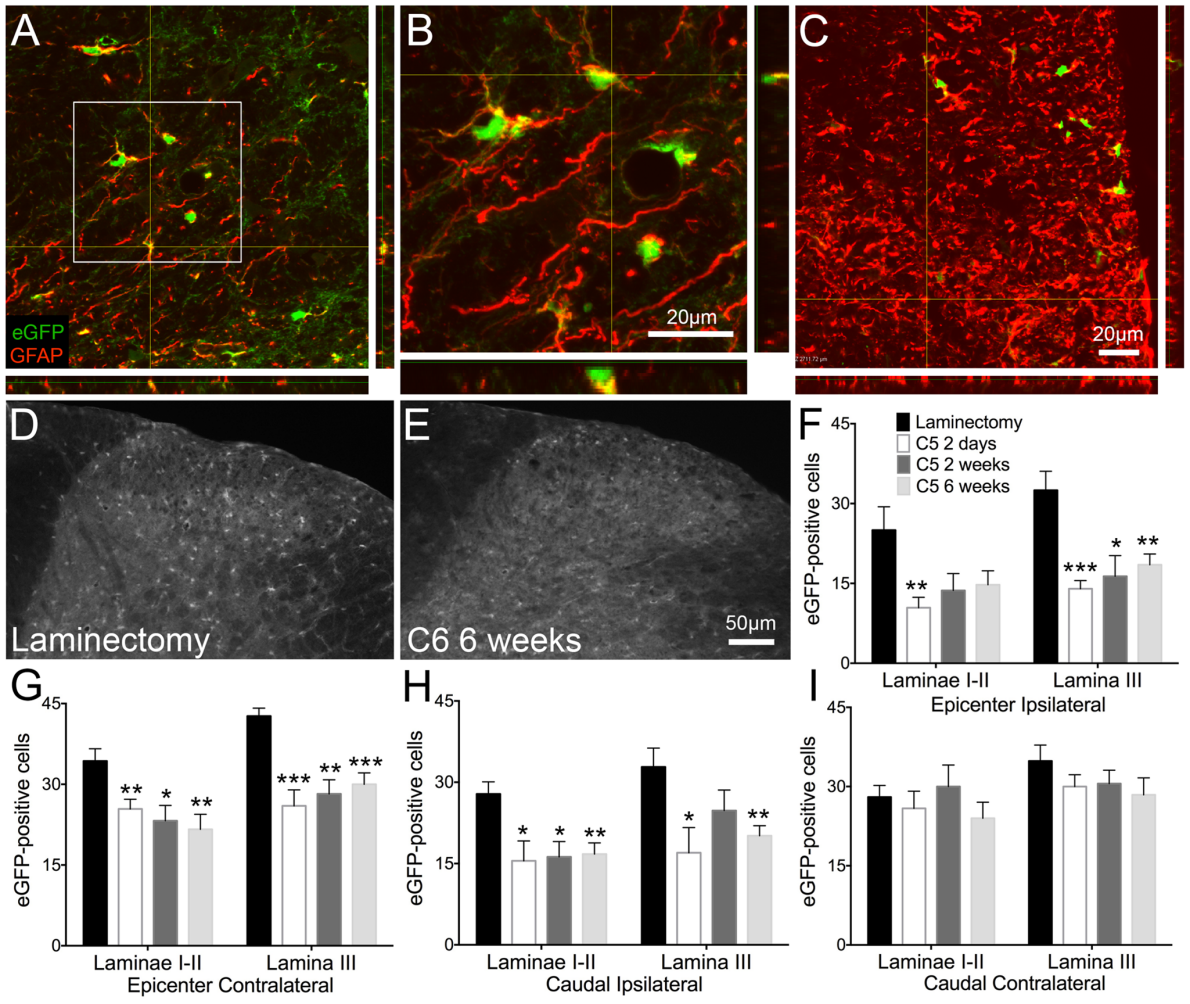
Astrocyte GLT1 promoter activity was reduced in injured BAC-GLT1-eGFP transgenic reporter mice. Following cervical contusion SCI, GLT1 expression/promoter activity, represented by eGFP-positive cells, was decreased in the superficial dorsal horn. In laminectomy animals, there were low levels of GFAP, although many of the cells that were GFAP-positive also expressed GLT1 (A–B). Following injury, however, an increase in GFAP was observed, but fewer of these activated astrocytes co-expressed GLT1 (C). Laminectomy control animals (D) had greater numbers of eGFP-positive cells compared to animals that received contusion injury two days, two weeks, or six weeks (E) prior to analysis. This decrease in GLT1 expression was observed at the injury epicenter in both the ipsilateral (F) and contralateral (G) superficial laminae as well as caudal to the injury on the ipsilateral side (H). No change in the number of eGFP-positive cells was found caudal to the injury on the contralateral side (I). * = p<0.05; ** = p<0.01; *** = p 0.001.

In BAC-GLT1-eGFP reporter mice, the GLT1 promoter drives expression of eGFP [27]. Following injury, we sacrificed animals at two days (n = 7), two weeks (n = 9), and six weeks (n = 8) and quantified eGFP-positive cells separately in laminae I–II and lamina III. We used one-way ANOVA to compare the number of eGFP-positive cells at each time point. In uninjured animals (Fig. 6D; n = 6), eGFP expression was robust and widespread throughout the grey and white matter. Furthermore, almost all of the GFAP-expressing astrocytes in these regions co-localized with eGFP-positive cells (Fig. 6A–B). eGFP-positive cells were decreased, however, at two days, two weeks, and six weeks (Fig. 6E) at various regions in the spinal cord. Despite the increase in GFAP expression after injury, there were few cells co-expressing eGFP and GFAP, suggesting a loss of GLT1 expression in activated astrocytes (Fig. 6C). At the injury epicenter, eGFP expression was downregulated at two days ipsilaterally (Fig. 6F) and at all time points contralaterally in laminae I–II (Fig. 6G). In lamina III, a decrease in eGFP-positive cells was observed bilaterally at the injury epicenter (Fig. 6F–G). Caudal to the injury, we report a decrease in eGFP at all time points in laminae I–II and at two days and six weeks in lamina III (Fig. 6H). No changes in the contralateral dorsal horn caudal to the injury were observed (Fig. 6I).

**Figure 7:**
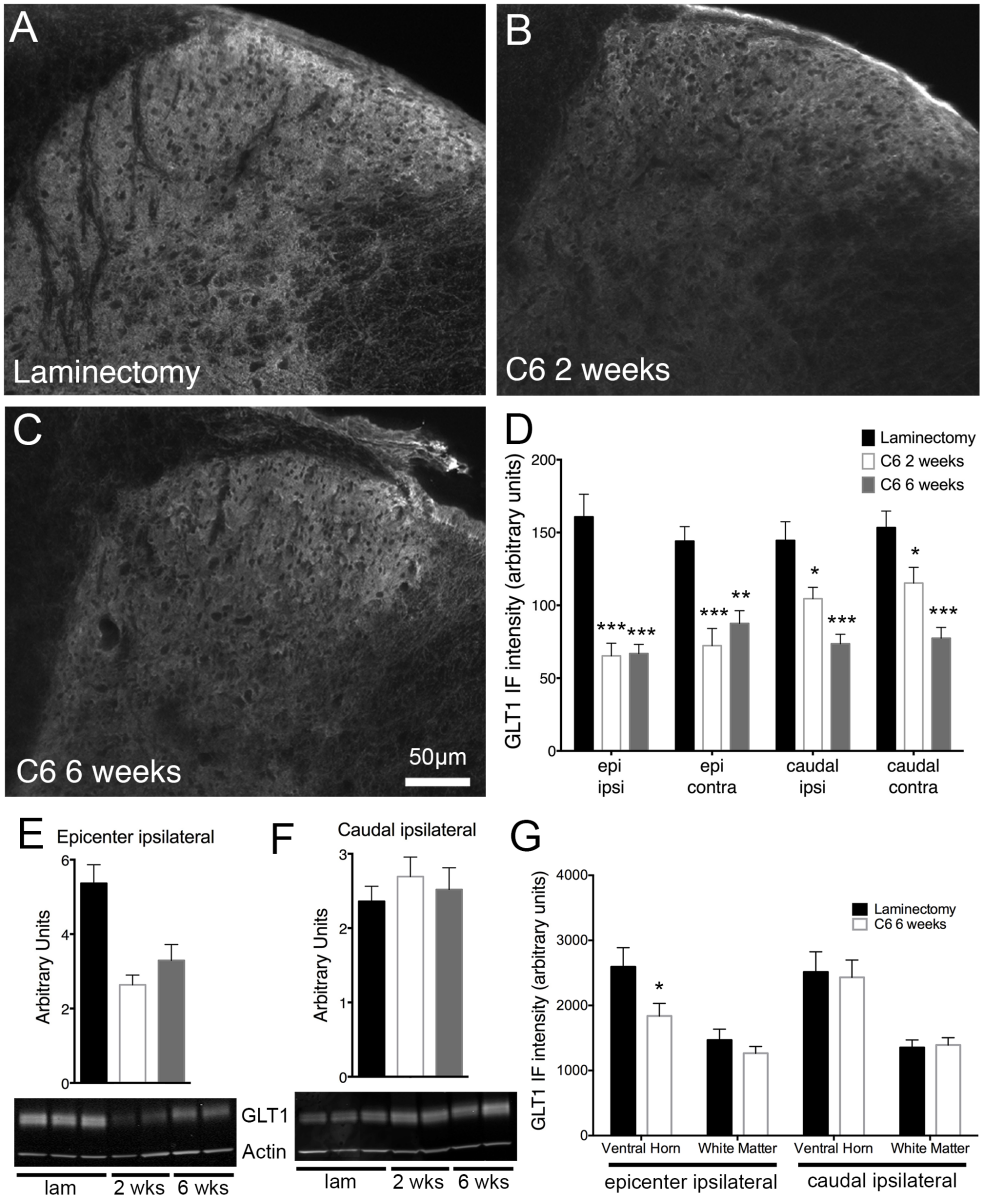
Cervical contusion SCI resulted in decreased astrocyte GLT1 expression in the dorsal horn. Immunohistochemical analysis revealed a decrease in GLT1 protein expression two (B) and six weeks (C) after cervical contusion SCI compared to laminectomy (A). This downregulation of the glutamate transporter was seen in the superficial dorsal horn on both sides of the spinal cord both at the injury site and caudal to the injury (D). Immunoblots of spinal cord tissue also showed a significant loss of GLT1 expression on the ipsilateral side at both time points following injury. However, this difference was seen only at the epicenter (E) and not caudal to the injury (F). Representative immunoblots are shown for each region (E–F). Analysis of GLT1 levels in regions of the spinal cord other than the superficial laminae at six weeks after injury reveal a loss of GLT1 expression in the ventral horn at the epicenter with no changes caudal to the injury (G). IF = immunofluorescence; epi = epicenter; ipsi = ipsilateral; contra = contralateral; lam = laminectomy; * = p<0.05; ** = p<0.01; *** = p 0.001.

In wild-type C57BL/6 animals, we employed immunohistochemical staining for GLT1 after C6 injury (n = 7–10) or laminectomy (n = 8). One-way ANOVA was used to identify significant differences in GLT1 staining between laminectomy, injured animals at 2 weeks, and injured animals at six weeks. Compared to laminectomy-only animals (Fig. 7A), animals receiving injury two weeks (Fig. 7B) or six weeks (Fig. 7C) prior to analysis showed a decrease in diffuse GLT1 protein expression in laminae I–II. These changes were seen at all regions analyzed (Fig. 7D). We further quantified the extent of GLT1 protein expression in injured (n = 7–8) and uninjured animals (n = 7) using immunoblotting techniques. At the injury epicenter, we observed a decrease in GLT1 protein in the ipsilateral hemicord at both time points after injury (Fig. 7E). Caudal to the injury, however, this glutamate transporter loss was not observed (Fig. 7F).

These data seem to contradict the immunohistochemical results we reported above. Thus, we performed immunohistochemical quantifications in regions outside of the superficial dorsal horn to try to identify the source of these contradictory results. At six weeks after injury, there is a significant loss of GLT1 expression in the ventral horn ipsilaterally at the epicenter compared to laminectomy animals (Fig. 7G). No significant change is seen in the ventral horn or white matter ipsilaterally caudal to the injury (Fig. 7G). These data suggest that, focally at the injury site, GLT1 loss occurs in multiple anatomical regions. However, caudal to the injury, the decrease in GLT1 expression may be more specific to the superficial dorsal horn (Fig. 7G). The changes in GLT1 from the immunoblotting data may not be evident due to the lack of anatomical specificity.

## Discussion

Here we describe changes in neuronal, astrocyte and microglial activation and GLT1 expression accompanying at-level thermal hyperalgesia in a cervical contusion SCI mouse model. In our model, robust and persistent thermal hyperalgesia and tactile allodynia are evident beginning one-two weeks after injury. Additionally, cervical contusion SCI induces an upregulation of ΔfosB, a marker of neuronal activity, in the superficial laminae of the dorsal horn. Astrocyte and microglial activation and proliferation, as measured by increases in GFAP, CD11b and Ki67 immunostaining, are also increased in these regions. We also observe a decrease in GLT1 expression and the number of GLT1-expressing cells in this model. These molecular changes that accompany hyperalgesia and allodynia in this study may contribute to the development of this aberrant pain response and represent important targets for better understanding the development of and treating neuropathic pain in SCI.

Hyperalgesia, defined as an increase in sensitivity to noxious stimuli, whether due to a decrease in pain threshold or an increase in response to stimuli above the pain threshold [36], is common in humans and is associated with sensory changes resulting from neuronal hyperexcitability [34], [35]. In mice, hyperalgesia in response to thermal stimuli can be measured using the Hargreaves test, in which a noxious infrared heat stimulus is placed under the paw and the paw withdrawal is recorded. In the models presented here, we observe thermal hyperalgesia in the ipsilateral forepaw beginning two weeks following cervical contusion SCI and persisting for the duration of the experiment. This is consistent with data from other groups, which report the development of hyperalgesia between two and three weeks after contusion SCI at various spinal levels in the mouse [37], [38] [39]. In the rat, our group and others observed thermal hyperalgesia as early as one week after cervical contusion injury [24], [40].

Like injured animals, laminectomy control animals show a decrease in withdrawal latency one week after surgery in the ipsilateral forepaw. This finding was unexpected, but not previously unreported. The development of post-laminectomy pain has been studied in the context of the extent of laminectomy, spinal deformation, and the loss of spinal stability following surgery [41], [42]. Researchers also showed that minimal laminectomy can induce transient hyperalgesic behavior at about one week after surgery [41]. While the cause of this pain behavior is unknown, it could reflect peripheral changes or spinal deformity rather than central sensitization or only transient central sensitization.

The ipsilateral development of thermal hyperalgesia in the mouse after SCI is consistent with the injury we see histologically. At the site of the injury, we observe a loss of ventral horn motor neurons and a disruption of normal tissue anatomy on the ipsilateral but not contralateral side. This suggests a disruption of pain transmission circuitry on the side of the injury, which may contribute to the hyperalgesia in the ipsilateral forepaw displayed by injured animals. The unilateral extent of the injury is also consistent with previous findings from our group; we previously reported damage to the ipsilateral dorsolateral and ventrolateral funiculi that did not extend bilaterally following moderate unilateral cervical SCI [10].

Unlike other studies [40], [44], we observe thermal hyperalgesia ipsilaterally but not contralaterally to the site of the injury. This discrepancy may reflect the modified version of the Hargreaves test that we utilized compared to these other groups. The Hargreaves test is most often used to measure thermal hyperalgesia in the hindpaw rather than the forepaw. Despite attempts to perform the Hargreaves test without restraint, including changes to the environment and longer acclimation times, we were unable to obtain reliable results. To overcome these difficulties, we restrained the mice in a way that did not impede forepaw movement but ensured that the mice remained still long enough to respond to the thermal stimulus. This method was introduced previously by Menendez, Lastra, Hidalgo, and Baamonde [28] for the study of hyperalgesia in the mouse forepaw. While the restraint improved the reliability of our thermal hyperalgesia measures, however, it may have affected the sensitivity of the mice to pain, which could have influenced the results we observed contralaterally.

Interestingly, while the hyperalgesia behavior changes we see are limited to the injured side of the spinal cord, we observe some molecular changes both ipsilaterally and contralaterally. ΔfosB and Ki67 expression is increased and GLT1 levels are decreased contralaterally after injury. The one alteration that is present ipsilaterally but not contralaterally is the activation of astrocytes. One possible explanation for the lack of hyperalgesic behavior in the contralateral forepaw is that the molecular changes we see are all required to elicit changes in pain behavior. There may be some interaction between activated neurons and glia that can disrupt pain transmission in the superficial laminae of the spinal cord following injury. Without activation of glia, pain transmission may continue normally.

Using von Frey filament testing, we assessed tactile allodynia following SCI. Allodynia, defined as a pain response to a previously non-noxious stimulus, is a common form of neuropathic pain [34]. In our model, we were able to identify allodynia following injury compared to baseline in the C6 injury group, but a similar significant effect was not observed in animals receiving C5 injury. We observed persistent tactile allodynia in both forepaws, which unlike the thermal testing results coincides with the anatomy of the histological findings.

Both hyperalgesia and allodynia are evoked pain behaviors in that they require a stimulus, whether noxious or non-noxious. We did not, however, study spontaneous pain, meaning pain that occurs in the absence of an external stimulus [43]. Spontaneous pain could potentially result, for example, from spontaneous activity in dorsal horn nociceptive neurons. Furthermore, while we measured at-level pain in the forepaw, we did not investigate below-level pain, which describes pain in dermatomes caudal to the level of injury and has been observed in the hindpaws of rats receiving cervical contusion SCI [40].

In this study, changes in pain behavior are exhibited in the absence of changes in motor behavior, as measured by grip strength. Because measures of evoked pain, such as the Hargreaves test and von Frey test, rely on motor activity (i.e. forepaw withdrawal) as a response, any dysfunction in motor behavior could affect test outcomes. The decrease in grip strength seen in the contralateral forepaw of C5 injury animals at one-week post injury was unexpected and could reflect transient local changes to the cervical spinal cord rather than a global alteration of pain transmission circuitry. Importantly, grip strength returns to baseline levels after this first week, at which point hyperalgesia and allodynia have developed.

Despite the maintenance of forepaw grip strength, we observed a significant loss of ventral horn motor neurons at the injury epicenter on the ipsilateral side following injury. One likely explanation for this disconnect is that our current injury model is milder and results in less motor neuron loss compared to our previous unilateral cervical contusion model in the mouse [9], which did produce persistent forelimb motor dysfunction. There is presumably some level of motor neuron loss necessary to result in quantifiable motor deficits.

At the molecular level, changes in ΔfosB and GLT1 were observed in the superficial dorsal horn of the injured spinal cord of our SCI model. Alterations in the expression of these proteins may contribute to central sensitization of pain transmission neurons in the cervical spinal cord. Central sensitization, including post-translational and transcriptional changes to dorsal horn neurons, affects the output of nociceptive neurons and the way in which peripheral stimuli are perceived. Thus, the observed changes may contribute to the hyperalgesic and allodynic phenotypes seen in our cervical contusion SCI.

C-fos is a member of the Fos family of transcription factors, which are induced quickly and transiently by a variety of stimuli. Because its expression is stimulated by an influx of calcium into neurons, c-fos acts as a marker of neuronal activity [45]. Previously, researchers have shown an induction of c-fos expression in the dorsal horn of the spinal cord following exposure to noxious stimuli in various forms [46],[47], [48], [49], [50]. ΔfosB is a truncated splice variant of the Fos family member, FosB. Unlike c-fos, which is expressed quickly but returns to basal levels within hours after stimulation, ΔfosB is induced gradually and is stable, so it accumulates over time and persists on the order of weeks or months [50]. ΔfosB has been implicated in a number of processes in the nervous system, including addiction, plasticity, and stress [45], [50] but may also play a role in neuronal changes associated with pain [14], [15].

In our SCI model, we see long-term elevation in the levels of ΔfosB in the superficial dorsal horn of the spinal cord, suggesting persistently increased activation of these neurons. These results are in accordance with another study that reported an increase in ΔfosB expression following pain induction, specifically carrageenan-induced inflammation [14]. Additionally, we previously reported an increase in ΔfosB expression in the rat following cervical contusion SCI [24]. While ΔfosB expression is significantly increased ipsilaterally at two weeks after injury, there is an even greater increase that spreads bilaterally at the six-week time point. This is consistent with the protein's stability and tendency to accumulate over time [46]. Furthermore, this persistent expression of the protein in the dorsal horn suggests an increase in the activity of these nociceptive neurons, possibly due to an increase in spontaneous firing and/or synaptic input, which may contribute to central sensitization. Thus the expression of ΔfosB that we see in our injury model is likely in accordance with its role in pain and plasticity.

In the nucleus accumbens and cortex, identified targets of ΔfosB include GluA2 and GluN1. In these regions, ΔfosB has been shown to upregulate these two proteins [50]. These targets are subunits of the glutamate receptors AMPAR and NMDAR, and their upregulation may contribute to increased glutamatergic transmission. Excessive stimulation of glutamate receptors can lead to hyperexcitability directly by increased activation of dorsal horn neurons and indirectly via glutamate excitotoxic loss of inhibitory interneurons [18]. It is unknown whether ΔfosB also targets these AMPAR and NMDAR subunits in the spinal cord following SCI. However, researchers have reported changes in GluA1 trafficking and expression in spinal neurons following central and peripheral injury and pain [51], [52], [53] as well as an upregulation of GluN1 and GluN2A in the ventral horn as a result of contusion SCI [54]. Despite this potential connection between ΔfosB and glutamate receptor subunits, ΔfosB does not definitively measure hyperexcitability. Instead, a continuation of this experiment to provide more conclusive evidence of the hyperexcitability could include electrophysiological measures of dorsal horn neurons.

Also following injury, we observe an increase in GFAP, a protein upregulated in activated astrocytes, in the dorsal horn of the spinal cord. We see this change at both time points, but it is more pronounced two weeks after injury. These data are in accordance with our group and others who have shown localized increases in markers of astrocyte activation following SCI [23], [24], [55], [56]. The observed increase in GFAP is seen only at the injury epicenter on the ipsilateral side, suggesting that this change is region-specific and may not play a role in potential above- or below-level changes, at least in our model.

The increase in activated astrocytes in our model of SCI could potentially have negative effects on the injured spinal cord and pain transmission circuitry. Often, activated astrocytes in the context of SCI and other injuries are considered detrimental because of their role in the formation of the glial scar, which can inhibit axon regeneration and plasticity, and the release of pro-inflammatory and other harmful molecules that can alter the excitability of neurons. Astrocytes, however, play many beneficial roles in the CNS, such as maintaining extracellular ion and neurotransmitter homeostasis, providing neurotrophic factor support, aiding in metabolic functions, and regulating the formation and maintenance of synapses [57].

Interestingly, while at-level thermal hyperalgesia is maintained through six weeks after injury, GFAP levels decrease slightly by this time point. Activated astrocytes play a significant role in the structural and molecular maintenance of the injury site [13], [17], [18], [57], [58]. The repair process following spinal cord injury, however, occurs in various phases, including acute, sub-acute, and chronic. While the effects of the glial scar and other repair processes can last months or years, not all of the molecules contributing to post-injury repair may be present at all stages. Thus, some astrocytes may return to the basal state in the days or weeks following injury, explaining the decrease in GFAP we see at six weeks compared to two weeks. Another explanation for the persistence of thermal hyperalgesia and tactile allodynia despite GFAP levels decreasing is that the loss of GLT1 also persists and contributes more strongly to the development of neuropathic pain after SCI. Alternatively, there may be irreversible damage to the spinal cord at this point that the inactivation of astrocytes may have no effect on.

Considering the various roles that astrocytes play following injury, it is important not only to study the reactive state of astrocytes in our model but also the effects of our injury on important homeostatic and protective functions of astrocytes. In light of this, we decided to investigate how our injury paradigm alters a centrally important function of astrocytes in the CNS, the uptake of glutamate through GLT1. Several groups have reported decreases in GLT1 levels following SCI [22], [23], [24], [25], [26], but the role of such changes has not been studied extensively in the context of neuropathic pain. To study GLT1 expression, we utilized both C57BL/6 wild-type and BAC-GLT1-eGFP reporter mice as well as both immunohistochemistry and western blotting. In our mouse cervical contusion SCI model, GLT1 levels are diminished in the dorsal horn at both time points at the epicenter and caudally on the ipsilateral side, suggesting a significant compromise in glutamate clearance in the injured spinal cord. Unlike with wild-type mice, in BAC-GLT1-eGFP mice, we see no change in the caudal contralateral region of the dorsal horn. This is likely due to a slightly different injury used in these two mouse paradigms.

Western blotting reveals that GLT1 protein levels are decreased ipsilaterally at the epicenter. However, no change in GLT1 levels is observed at the caudal ipsilateral region, which is contrary to the results we report using immunohistochemistry. This discrepancy likely reflects the difference in spatial resolution between these two techniques. Using immunohistochemistry, analyses of very specific regions, such as the superficial dorsal horn, are possible. For western blotting, however, we use a piece of tissue that encompasses an entire spinal cord level, including both the dorsal and ventral horn and surrounding white matter tracts. For our immunohistochemical analyses of the injured spinal cord, we examined the most superficial laminae of the dorsal horn, laminae I–II, as well as laminae III.

Importantly, co-localization analyses of GFAP and GLT1-eGFP at high-magnification reveal that, in control animals, most of the GLT1-positive cells are also GFAP-positive astrocytes. However, after injury, while the number of GFAP-positive cells is increased, the number and percentage of these astrocytes also expressing GLT1 is greatly reduced. This suggests that the loss of GLT1 that is observed in our animals occurs mostly in astrocytes, supporting our proposal that astrocytes activated after injury are deficient in glutamate clearance.

Like astrocytes, microglia play a major role in both immediate and delayed responses to injury to the spinal cord and may contribute to the development of pathological pain [13]. We also noted significant microglial activation in the superficial dorsal horn following cervical contusion SCI. Unlike astrocyte reactivity, we observed microglial activation not just at the ipsilateral epicenter region, but also at contralateral epicenter and caudal ipsilateral locations. Though we focused mostly on astrocyte changes, including down-regulation of GLT1 expression, it is likely that microglial activation also played a significant role in the persistent neuropathic pain phenotype observed in this study.

Here we present a model of unilateral cervical contusion SCI in the mouse that is representative of typical injury seen clinically in humans [1], [5]. The model exhibits at-level neuropathic pain accompanied by relevant molecular changes in the superficial dorsal horn. The protein expression changes suggest the presence of activated and proliferating astrocytes and a loss of GLT1 function, which may contribute to central sensitization, and underlie the development of neuropathic pain. We have identified a number of proteins that are altered by SCI and accompany thermal hyperalgesia and tactile allodynia, but their roles in the development of neuropathic pain have yet to be fully elucidated. Further research with the model may include identifying the factors and processes underlying the formation and maintenance of neuropathic pain in the spinal cord as well as recognizing targets for the treatment of chronic pathological pain resulting from SCI and similar injuries. Importantly, the availability of this model in the mouse allow for the use of valuable transgenic tools.
